# Nonunion of an Isolated Medial Condyle of a Femur Hoffa Fracture in a 21-Year-Old Male: A Case Report

**DOI:** 10.7759/cureus.44722

**Published:** 2023-09-05

**Authors:** Ravindra Mohan, Rishabh Agarwal, Mohammad Baqar Abbas, Sanjiv Kumar, Arpit Singh

**Affiliations:** 1 Orthopedic Surgery, King George's Medical University, Lucknow, IND; 2 Orthopedic Surgery, Jawaharlal Nehru Medical College, Aligarh, IND

**Keywords:** trauma, femur, hoffa fracture, medial condyle, non union

## Abstract

Isolated Hoffa fractures of the femur are often missed on initial radiographic evaluations. Routine CT scans for intraarticular fractures in suburban populations are not routinely done. Nonunion of medial condyle Hoffa fracture of the distal femur is a rare finding and presents late with pain in weight-bearing and painful flexion. This is a case report of a 21-year-old male who sustained trauma as a result of a motorcycle-car collision and was managed elsewhere conservatively on an above-knee slab. He presented after five months to our side with a limited range of movement at the knee and pain during ambulation. After radiological work diagnosis of isolated non-union of medal condyle Hoffa fracture of the distal femur was made, the patient was managed by freshening of fracture followed by rigid fixation with cancellous screws and reconstruction plate. At postoperative six weeks, the patient had a painless full range of motion at the knee joint.

## Introduction

Commonly encountered fractures of the distal femur are in the axial and sagittal planes. A Hoffa fracture (33-B3.2 as per the AO/OTA classification) is a coronal split fracture that can be appreciated in the lateral view X-rays of the distal femur [1]. The mechanism of a Hoffa injury is poorly understood, and most articles point to axial loading force in the hyper-flexed knee with an adduction and internal rotation component leading to medial condyle Hoffa fracture [2,3]. Being intra-articular fractures, absolute reduction and restoration of joint congruity are mandatory as with other intra-articular fractures. The strong pull of the gastrocnemius muscle and combined forces of the adductor magnus, quadriceps, and hamstring around the distal femur necessitates open reduction and secure fixation. Human knees are in physiological valgus; therefore, an isolated medial condyle Hoffa fracture with an intact lateral femoral condyle is an extremely rare type of injury and has not received adequate mention in the literature in comparison to other intra-articular fractures [4-6].

## Case presentation

**Figure 1:**
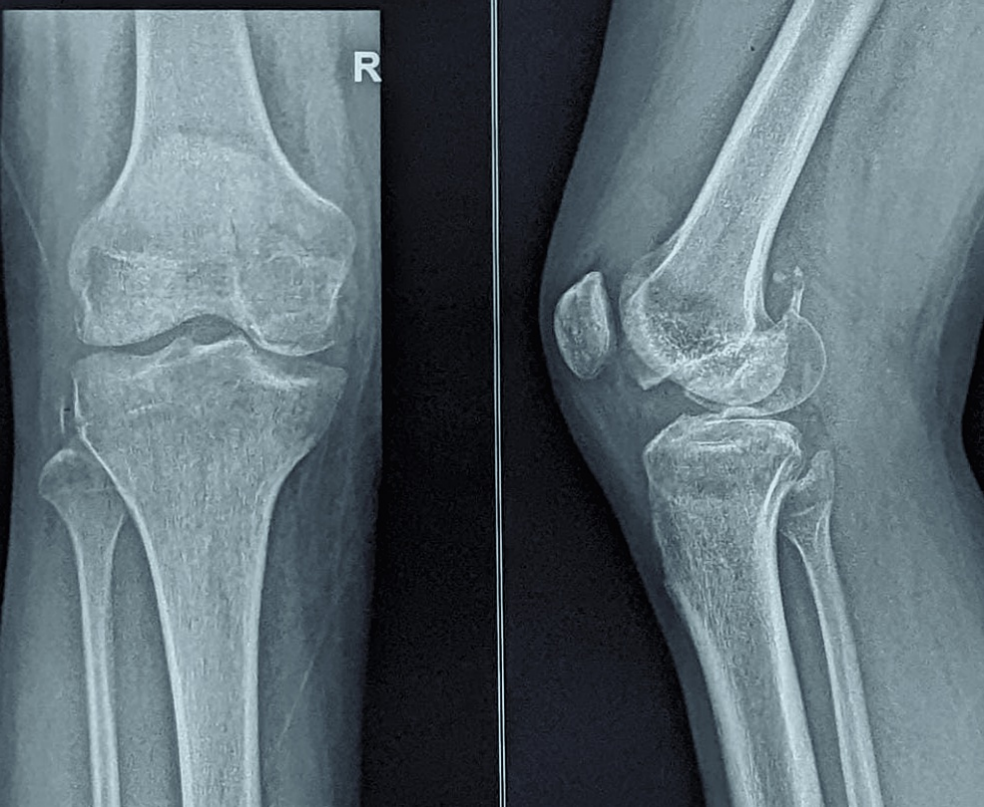
Plain radiograph of the right knee in anteroposterior and lateral view showing the Hoffa fracture of the medial condyle distal femur

**Figure 2:**
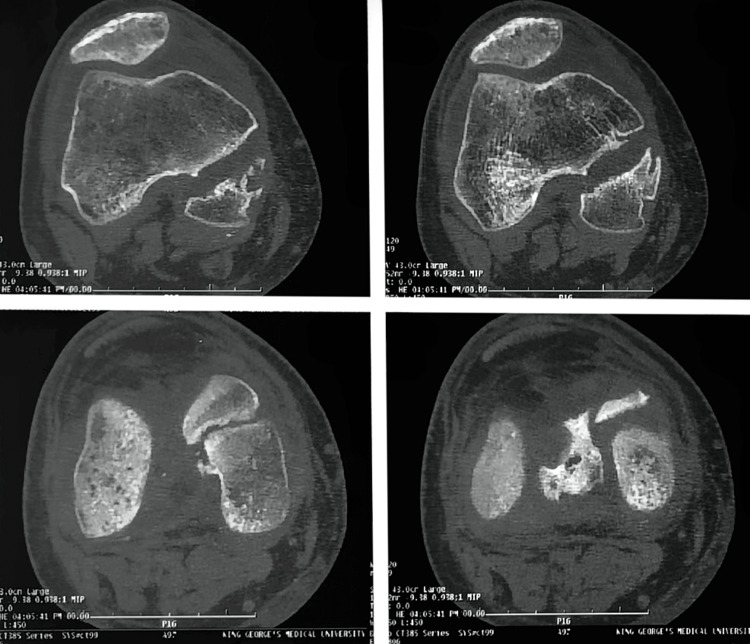
CT showing the medial condyle Hoffa fracture (R)

**Figure 3:**
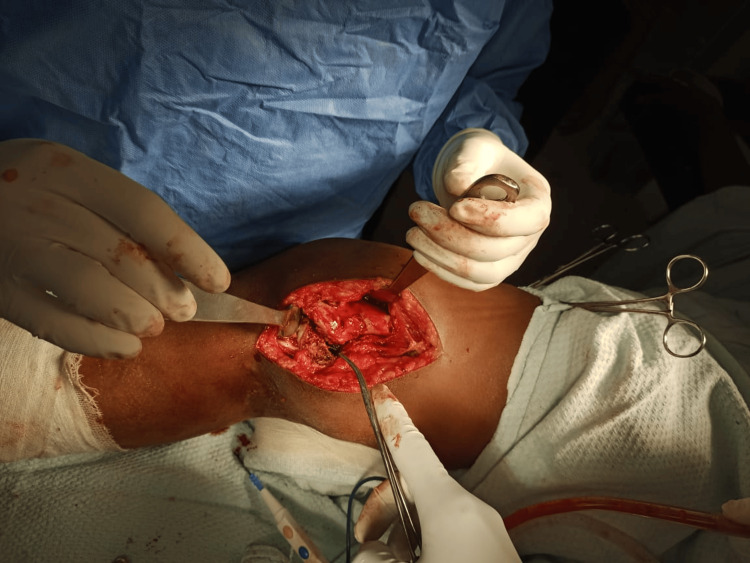
Intraoperatively fibrous tissue is seen between the fracture ends

**Figure 4:**
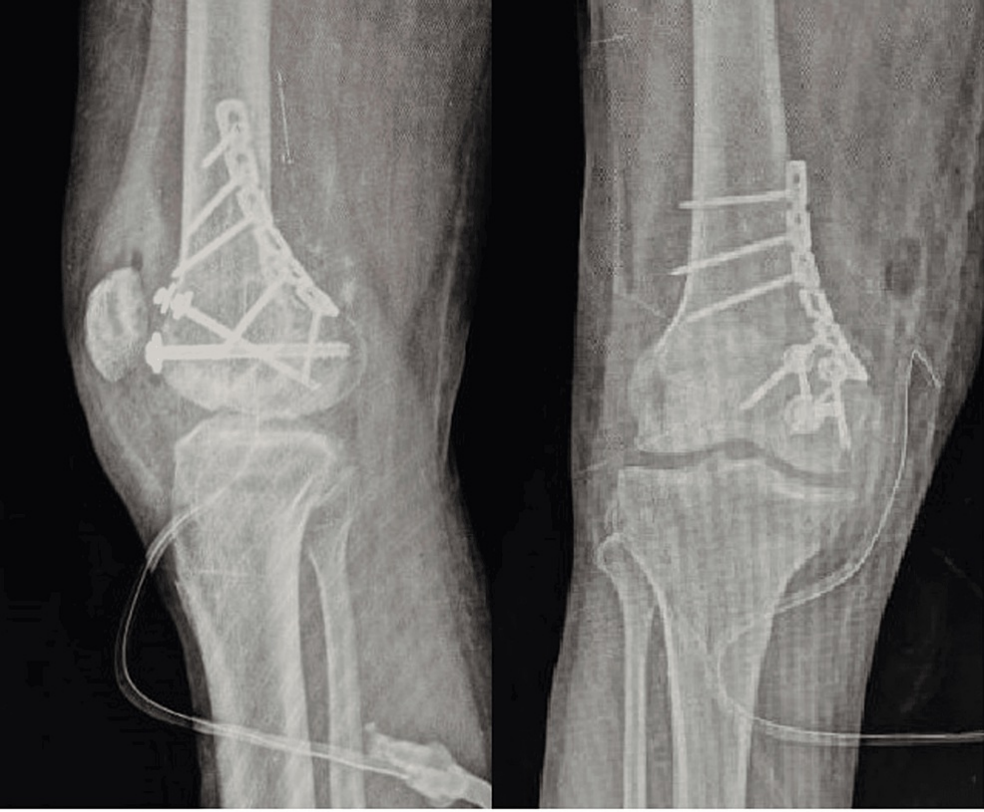
Postoperative X-ray

**Figure 5:**
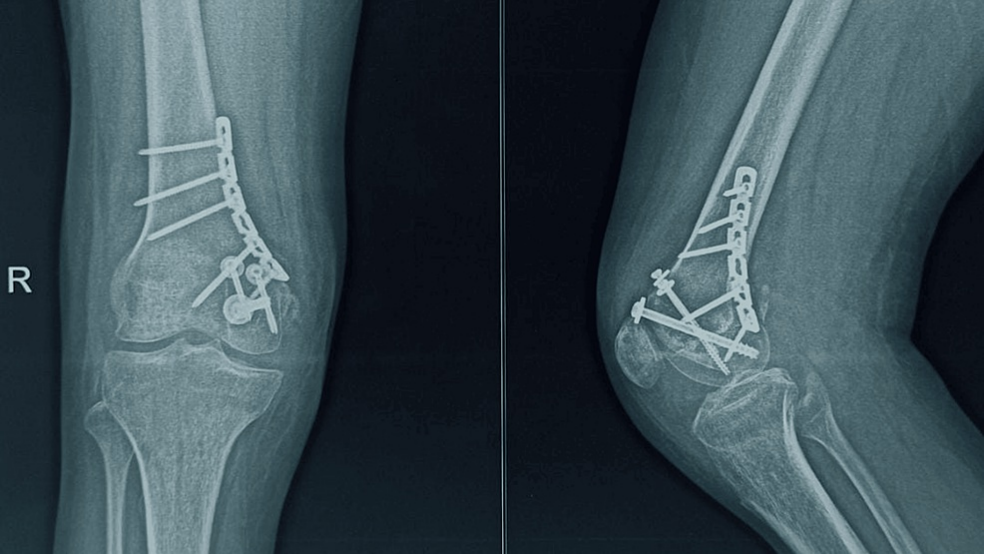
Postoperative at six weeks

**Figure 6:**
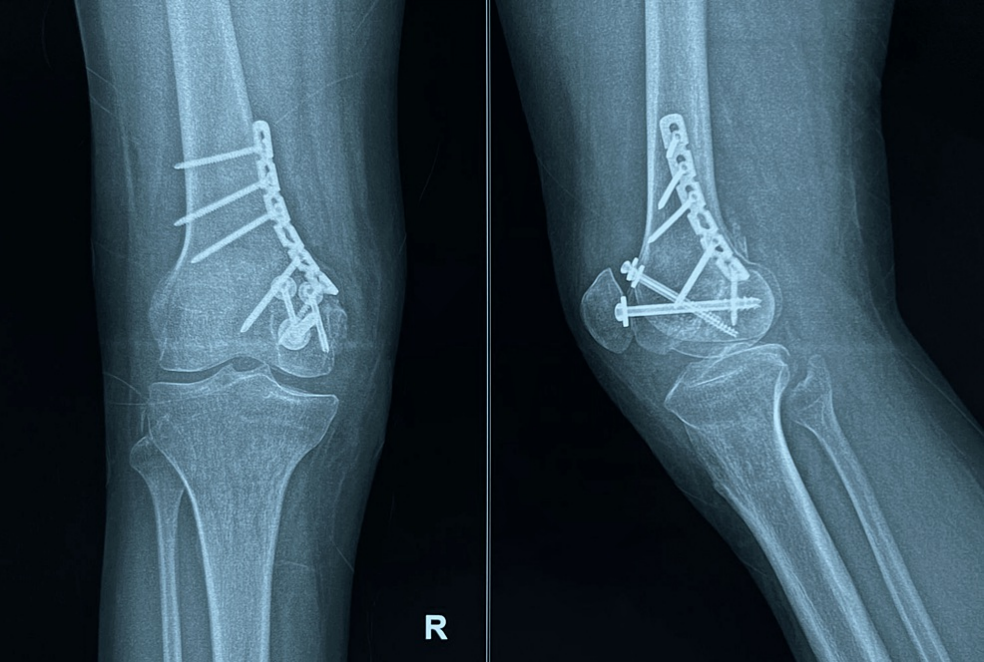
Postoperative at 5 months showing union

**Figure 7:**
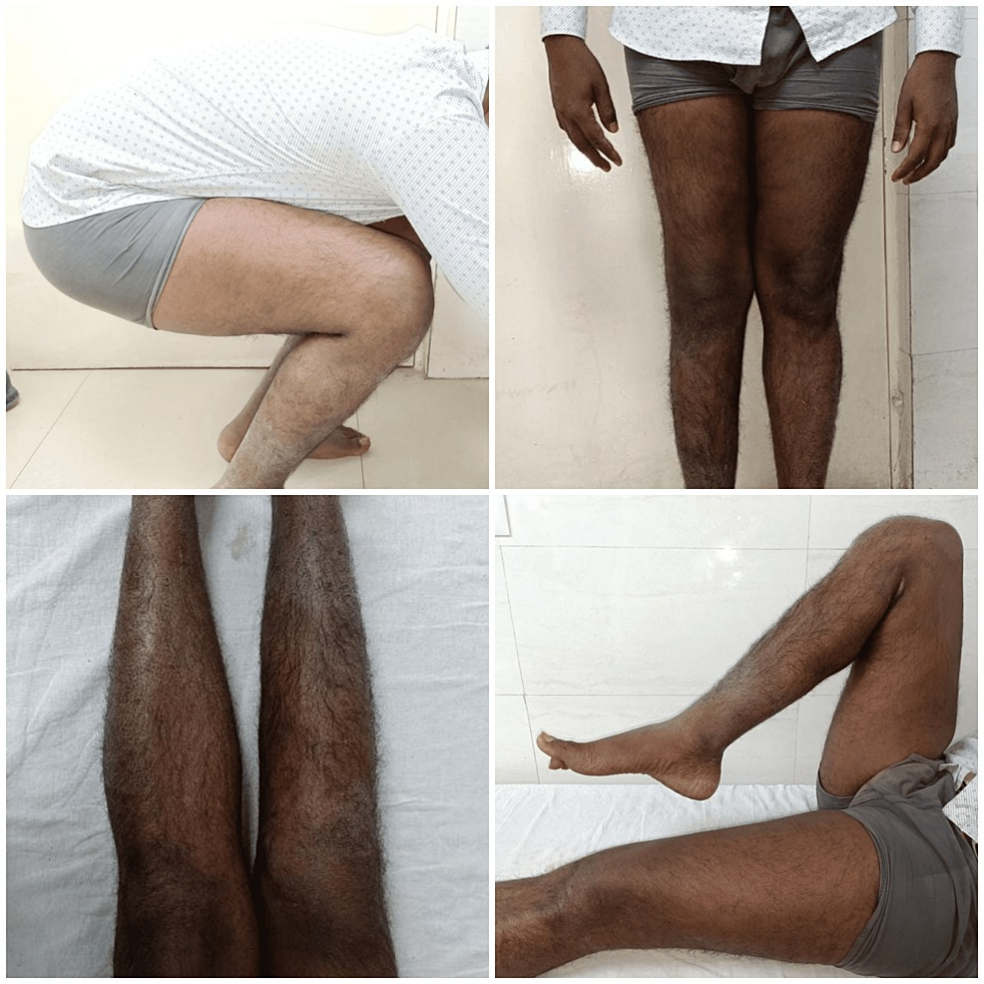
Excellent range of motion postoperative at six weeks

A 21-year-old male presented to our outpatient department with a complaint of pain over (R) his knee and decreased range of motion. He sustained trauma five months before presentation as a result of a motorcycle-car collision following which he consulted a local practitioner. The fracture was missed on initial X-rays, but he was put on above knee slab for three weeks and prescribed some painkillers. On removal of the slab, he had difficulty bearing weight and could not fully extend the knee. On presentation, his range of motion was 10o to 70o with flexion deformity of 10o. Anterior-posterior and lateral X-rays revealed a non-united Hoffa medial condyle femur (R) type III (Letenneur classification) (Figure 1). Following a CT scan, the patient was planned for fixation (Figure 2). On the supine position with the knee slightly flexed in the figure four position, a six cm incision was given in the line of the tendon of the adductor magnus. Fracture ends were opened and freshened (Figure 3). An iliac crest graft was taken and designed to create the anatomy of the medial condyle and was secured with 2 K wires. One 6.5 mm and two 4 mm cannulated cancellous screws with washers were used to secure the fracture, and the k wires were then removed (Figure 4). The knee was taken to the complete range of motion and partial weight bearing from the next day on a knee ROM brace. After stitch removal, the knee flexion was gradually increased, and at the six-week follow-up, it was from full extension to about 120o flexion (Figure 5). At the five-month follow-up, the fracture was completely united and pain-free, and the knee ROM was from full extension to 130o of flexion (Figures 6-7).

## Discussion

The most important finding of the case was the importance of initial evaluation of the patient as it can significantly decrease the morbidity of the patient. CT scans are to be routinely advised for such fractures as they may be easily missed on routine radiography, and they also help in planning fixation [7,8]. The lateral condyle Hoffa is more common due to the natural valgus of the knee joint and is, therefore, three times more common than its medial counterpart [9]. Late presentation and initial loss of treatment account for the major cause of nonunion in such fractures [10]. Being intraarticular fractures, conservative treatment often fails to give union. Lewis et al. observed that seven cases of Hoffa fractures and two patients treated conservatively did not yield a good outcome [11]. Jiang et al. [12] treated the nonunion of a Hoffa fracture for a 27-year-old male with internal fixation and used a xenogenous bone graft with excellent results. Pai Manjeswar et al. [13] reported a similar case report, and they achieved excellent results with internal fixation but did not use bone graft. The interposition of soft tissue between fracture ends contributed to nonunion in our patient. The use of a reconstruction plate and gradual range of motion on the ROM brace was advised to support our fixation from the strong pull of the gastrocnemius muscle. Long-term follow-up of the patient is excellent. He is pain-free and has returned to his job.

## Conclusions

Union with a good range of motion could be achieved at the end of five months in our case. Medial condyle distal femur Hoffa fracture though rare should not be missed at initial evaluation. Once the knee is pain-free, a thorough examination to look for ligamentous injuries should be undertaken. The surgical aim is to provide for an anatomical reduction and a secure fixation. The use of a bone graft sandwiched between the fracture ends and a neutralization plate to support the fixation is an excellent way to restore the knee geometry.
